# Dr. Bushrod W. James

**Published:** 1903-02

**Authors:** 


Dr. Bushrod W. James, of Philadelphia, a prominent homeopathic practitioner and author, died at his residence, January 7, 1903, aged 67 years.

